# Foreign Body in Duodenum Mimicking a Duplication Cyst on Imaging

**DOI:** 10.21699/ajcr.v7i5.468

**Published:** 2016-11-01

**Authors:** Aditya Pratap Singh, Vinay Mathur, Ramesh Tanger, Arun Gupta, Ayush Kumar

**Affiliations:** Department of Pediatric Surgery, SMS Medical College Jaipur, Rajasthan, India

**Keywords:** Crystal gel ball, Duodenum, Duplication, Foreign body

## Abstract

Paediatric age group is most vulnerable for the accidental foreign body (FB) ingestion which may go unnoticed. These patients present with symptoms or complications as a result of FB and may mimic other conditions on various investigations. We describe a 9-month old infant who ingested crystal gel ball and presented with vomiting for a month. On radiological imaging it was interpreted as duplication cyst of the duodenum. At operation, crystal gel ball was retrieved. Our case vindicates importance of keeping various possibilities in mind as differential diagnoses during evaluation and management of surgical ailments such as the duplication cyst of duodenum.

## CASE REPORT

A 9-month-old female child presented with a history of intermittent vomiting for a month. The vomitus was generally non-bilious but occasionally bilious but she had been taking orally during this period. There was no significant past history. On examination, abdomen was soft and non-distended. All routine blood investigations were in normal limits. X-ray abdomen showed dilated stomach. On upper gastrointestinal contrast study, a filling defect in the duodenum with dilated stomach was noted. Ultrasonography abdomen (USG) suggested a cystic structure in relation to the duodenum (Fig. 1). Contrast enhanced computerised tomography (CECT) depicted a hypodense structure in relation to duodenum and suggested duplication cyst as the most probable diagnosis (Fig. 2). On exploration, there was dilated stomach and duodenum up to D2, but no duplication cyst could be found. On palpation of duodenum, there was a firm intraluminal structure with no external sign of the any pathology. We broke the foreign body intraluminally with fingers and squeezed it distally to remove it per rectally in piecemeal (Fig. 3). It was crystal gel ball and which swelled up in duodenum. On further inquiry, mother admitted purchase of crystal gel balls for decorative purpose. The patient was discharged after 7 days and doing fine on follow-up.

**Figure:**
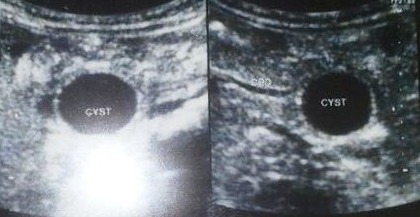
Figure 1:USG showing a hypoechoic cyst looking area in relation to duodenum.

**Figure:**
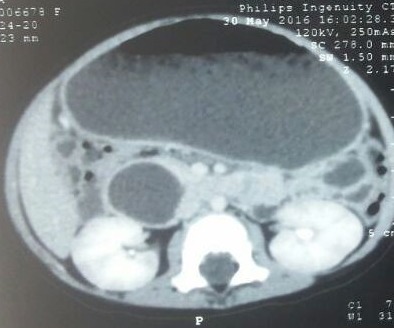
Figure 2:CECT showing hypodense area in relation to duodenum. Stomach is also hugely dilated.

**Figure:**
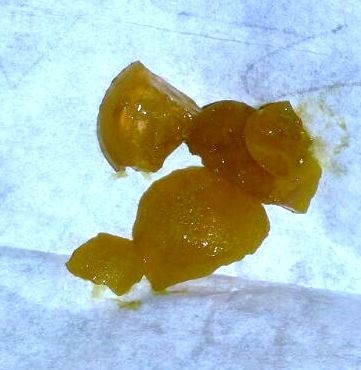
Figure 3:Per rectally removed parts of the crystal gel ball.

## DISCUSSION

Crystal gel balls are composed of super absorbent polymer (SAP) with the property of water absorption and thus they increase in size. Its ingestion can lead to various complications, from partial to complete intestinal obstruction. It may result in intestinal perforation and peritonitis.[1-3] In our case it caused partial duodenal obstruction for a month.

Decorative crystal jelly balls are radiolucent. Radiograph will only show the complications of foreign body ingestion.[1] Problems arise when history of FB ingestion is not given. Faizah et al showed that an intraluminal crystal jelly ball could mimic other sonographic pathologies, such as an enteric duplication cyst.[3] In our case crystal gel ball had occupied duodenum and its hypoechoic shadow on USG and hypodense nature on CT scan simulated a cystic structure in relation to duodenum thus mimicked as duplication cyst. Community awareness is mandatory to stop using such material in homes as a case of mortality secondary to ingestion of crystal gel ball ingestion has been reported in literature.[2]

## Footnotes

**Source of Support:** Nil

**Conflict of Interest:** None declared

